# Academic leagues: a Brazilian way to teach about cancer in medical universities

**DOI:** 10.1186/s12909-015-0524-x

**Published:** 2015-12-30

**Authors:** Diogo Antonio Valente Ferreira, Renata Nunes Aranha, Maria Helena Faria Ornellas de Souza

**Affiliations:** 1 School of Medical Sciences, University of State of Rio de Janeiro, Rua Almirante Baltazar n 189 apt 513, São Cristovão, Rio de Janeiro, RJ CEP: 20941-150 Brazil; 2 Department of Gynecology and Obstetrics, School of Medical Sciences, University of State of Rio de Janeiro, Boulevard 28 de setembro, n77, 5 floor. Vila Isabel, Rio de Janeiro, RJ CEP: 20550-170 Brazil; 3 Department of Pathology and Laboratory, School of Medical Sciences, University of State of Rio de Janeiro, Avenida Professor Manuel de Abreu n 444 – 4 floor. Maracanã, Rio de Janeiro, RJ CEP: 20550-170 Brazil

**Keywords:** Medical education, Oncology, Academic leagues, Cancer education, Students’ skills

## Abstract

**Background:**

Performance of qualified professionals committed to cancer care on a global scale is critical. Nevertheless there is a deficit in Cancer Education in Brazilian medical schools (MS). Projects called Academic Leagues (AL) have been gaining attention. However, there are few studies on this subject. AL arise from student initiative, arranged into different areas, on focus in general knowledge, universal to any medical field. They are not obligatory and students are responsible for the organizing and planning processes of AL, so participation highlights the motivation to active pursuit of knowledge. The objective of this study was to explore the relevance of AL, especially on the development of important skills and attitudes for medical students.

**Methods:**

A survey was undertaken in order to assess the number of AL Brazilian MS. After nominal list, a grey literature review was conducted to identify those with AL and those with Oncology AL.

**Results:**

One hundred eighty of the 234 MS were included. Only 4 MS selected held no information about AL and 74.4 % of them had AL in Oncology. The majority had records in digital media. The number of AL was proportional to the distribution of MS across the country, which was related to the number of inhabitants.

**Conclusions:**

The real impact and the potential of these projects can be truly understand by a qualitative analysis. AL are able to develop skills and competencies that are rarely stimulated whilst studying in traditional curriculum. This has positive effects on professional training, community approach through prevention strategies, and development on a personal level permitting a dynamic, versatile and attentive outlook to their social role. Besides stimulating fundamental roles to medical practice, students that participate in AL acquire knowledge and develop important skills such as management and leadership, entrepreneurship, innovation, health education, construction of citizenship. Oncology AL encourage more skilled care to patients and more effective policies for cancer control.

## Background

The global impact of cancer is increasingly significant. Recent data shows an approximate number of 14 million new cases with 8 million cancer-related deaths in 2012, affecting people of all countries [1]. Brazilian Cancer Institute estimates that 576,000 people will be diagnosed with a malignant disease in 2014 and 2015 [2]. Thus, the performance of qualified professionals, committed to cancer care (prevention, early diagnosis and screening) is critical. Recently, studies have highlighted the experiences of several training centers [3–8] and emphasized the importance about teaching and learning methodologies.

In Brazil, an initiative has recently been gaining attention on medical education. Several projects, called Academic Leagues (AL), emerged in medical schools around the country. In general, AL are arranged into different areas, such as Cardiology, Neurology and Oncology. Focus is not on an early specialization, but on a general view of knowledge that should be universal to any medical field. They are extracurricular activities, so participation highlights the students' motivation to active pursuit of knowledge [9]. Meanwhile, there are few studies about how AL impact medical education. AL are similar to the Learning Communities found in American and Canadian universities [10]. However, there are some specific particularities that should be discussed. In AL, students are responsible for the organizing and planning processes, with the assistance. The methodology of AL is organized into three main areas: learning, research and community education [11]. The activities occur regularly throughout the curricular semester, usually in unusual schedules, involving students from different years and even from different medical universities. AL frequently hold regional and national events, seminars and conferences, such as Brazilian Congress of Cancer, Congress of Oncology Leagues and Brazilian Congress of Medical Education, which promote scientific development and enable the exchange of experience between peers.

In this manuscript, we present a survey of the Oncology Leagues in Brazil and a critical reflection on the potential of these projects in Medical Education.

## Methods

A survey of all the medical schools (MS) officially registered in the Brazilian Ministry of Education database was conducted in June 2014. This research follows the approval of the Research Ethics Committee of Pedro Ernesto University Hospital, under the number 102.679.

### Inclusion criteria

MS that started curricular activities before 2010. This criteria was adopted considering the Brazilian MS curriculum, which consists of a total of six years, divided into two phases: theoretical and practical content (from the 1st to the 4th student year) and supervised internship (full-time during the last 2 years of the course). Thus, there was a guarantee that all selected institutions were categorized into active theoretical and practical modules.

### Exclusion criteria

MS that were inactive or in the disqualification process at the time of the survey.

### Analysis

We conducted a descriptive study, with results presented through percentages. The total MS that fulfill the inclusion and exclusion criteria was lifted. After a nominal ratio of MS, a grey literature review [12] was conducted in order to identify those with AL and those with Oncology Academic Leagues. A grey literature review is a research methodology widespread in social sciences that includes both published and unpublished materials, which are not identifiable by conventional literature review [13], for example: books, conference abstracts, reports, unpublished dissertations, policy documents and personal correspondence. [14] Articles, reports from scientific conferences, political-pedagogical projects and medical schools’ websites were analyzed. Searched engines such as Google® were used, and blogs, social network profiles and forum discussions in virtual communities were consulted. The territorial distribution of MS and LA and even the existence of inter-institutional projects was also considered in the analysis.

## Results

**Fig. 1:**
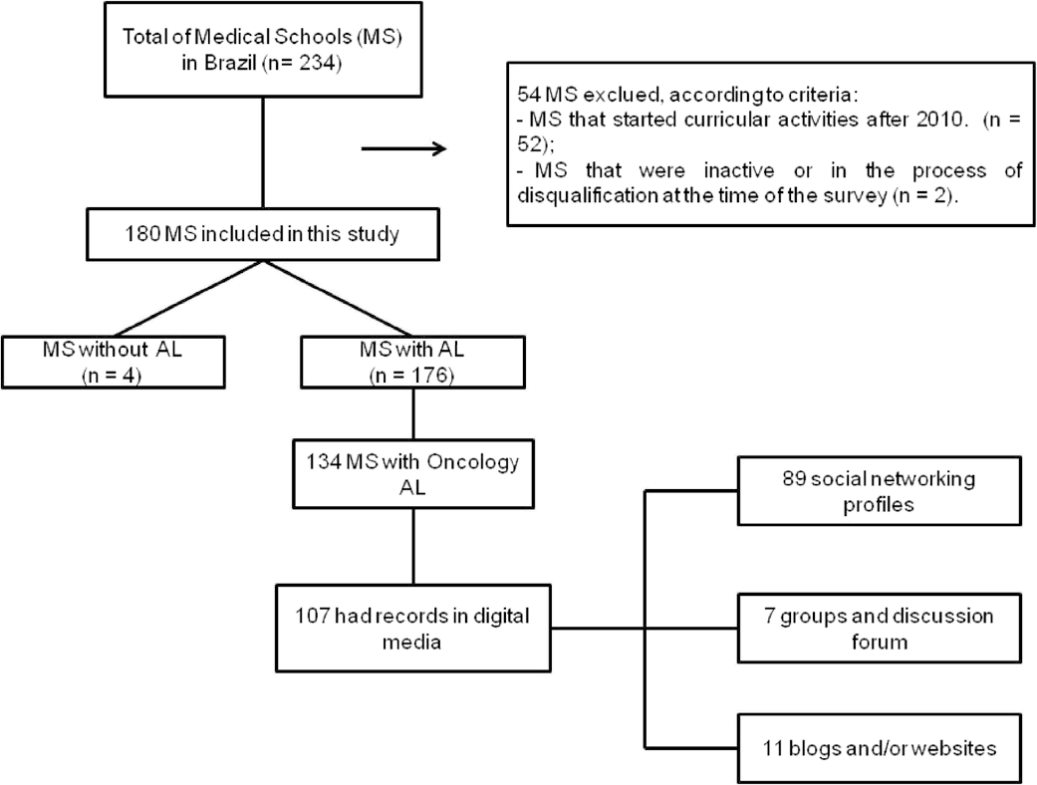
Flow diagram of study results

A hundred eighty were selected from the 234 MS officially registered in Brazil (Fig. 1).

Fifty four schools were excluded (52 began curricular activities after 2010 and two were in process of disqualification). Only four MS selected have no information about AL. From the schools with AL, 74.4 % of them had AL in Oncology. Most of them (107/134; 79.8 %) had records in digital media: social networking profiles (89/307; 83.1 %), blogs and/or websites (11/107; 10.2 %), groups and discussion forum (7/107; 6.5 %). Students from more than one MS undertaking the same project were involved in 16 Oncology AL. Therefore students from two MS without AL could engage in Oncology Leagues from other schools.

**Fig. 2:**
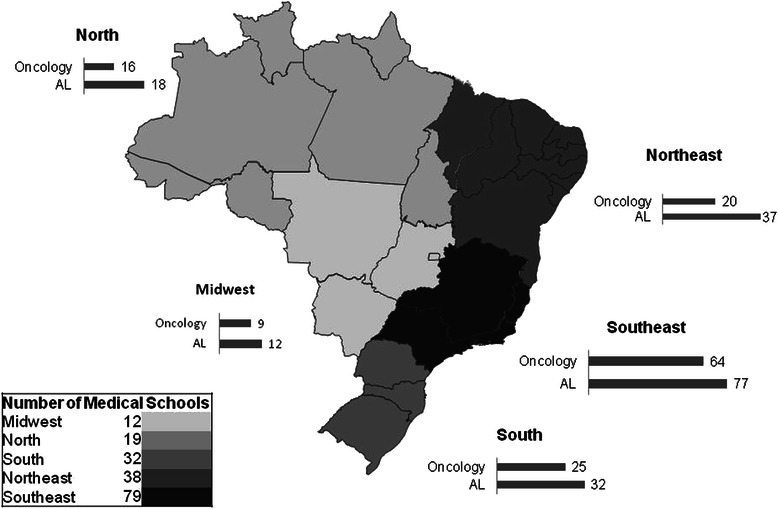
Distribution of medical schools (MS), academic leagues (AL) and oncology academic leagues (Oncology), for regions, in Brazil

Figure 2 illustrates AL distribution. Most of them are located in the Southeast, which is the main region of development and population in Brazil, constituting to 43.8 % of the total MS. The number of AL is proportional to the distribution of MS across the country, which is related to the number of inhabitants. All MS in the South and Midwest regions have AL. With regards to Oncology AL, it can be seen that the distribution is similar in the majority of the Brazilian regions (84.2 % in the North, 75.0 % in the Midwest, 81.0 % in the Southeast and 78.1 % in the South). In spite of being the second most populous region, the Northeast it is also the poorest and reached 52.6 %. Seventy five MS (43 %) were administered by Brazilian's government organizations and 105 (57 %) were managed by private organizations. AL were present in all of MS run by the government and in 96.2 % of MS managed by private organizations. There was no significant differences between the two MS with Oncology Leagues which amounted to 75 % and 77.3 %, respectively.

## Discussion

Similar to the increasing number of Learning Communities, [10] our results show the importance of AL in medical education in Brazil. However, the real impact and the potential of them could only truly understand by undertaking qualitative analysis of their activities. Firstly, AL are not mandatory activities, revealing the element of choice and self-motivation to pursue personal academic paths in interesting areas. Similar to the CanMEDS project and the skills described in the European Academy of Teachers in General Practice and Family Medicine (EURACT), [15, 16] all of the seven roles seen as fundamental to medical practice are developed by students that participate in AL (Table 1).

**Table 1:** Seven essential roles for the medical professional

	Description	AL contribution
Medical Expert	Apply knowledge, clinical skills and professional attitudes in the provision of patient-centered medical care.	Discussion of relevant health problems in general, humanistic and critical stance, based on the best scientific evidence.
Health Advocate	Use of expertise to advance health and welfare of patients, populations and communities, contributing to the reduction of inequalities in health.	Approach of subjects in context with people's health problems demands. Discussion of health promotion and disease prevention.
Manager	Participation in the organization of health care and contributes to the effectiveness of the health system.	Practical training in managing people (students members), projects and problems, bringing security to professional practice.
Communicator	Effective communication skills, facilitating the establishment of sound relationships with patients, families and colleagues.	Development of leadership and communication skills. Training for problem solving.
Collaborator	Optimal medical care through effective work in a health care team.	Multidisciplinary. Practice teamwork among members, sharing ideas from a common motivation.
Scholar	Lifelong commitment to learning, as well as the creation, dissemination, application and translation of medical knowledge through popular education in health,	Contact with research and methodologies that stimulate learning for life and constant updating.
Professional	Exercise ethical and sustainable practice and is consistent with their personal values and contributing to professional regulation.	All work process starts on students motivation: planning, scheduling, production and application of AL activities.

**Fig. 3:**
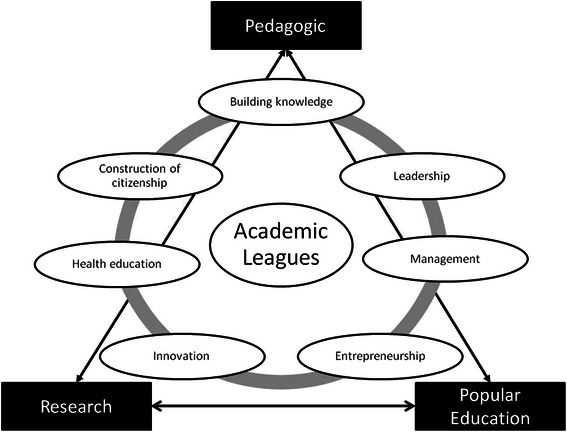
Skills developed through participation in Academic Leagues in three fields of university activities

Figure 3 represents all the skills developed by students that participate in AL.

### Building knowledge

AL address specific areas of medical knowledge in a multi-disciplinary way. Students are empowered to act against major local health problems, using the latest scientific advances and have constant contact with research. Beyond this, they became stimulated to participate in scientific meetings. Unlike traditional methods of teaching, AL can stimulate rapid acquirement of scientific knowledge because students are encouraged to learn how to learn and, thus, reinforces continuous learning, which is fundamental to professional life [17]. In fact, it was shown that involvement in research activities contributes to the development of skills in scientific methodology, critical appraisal, time management and teamwork that will influence patient care and professional training in the future [18], consolidating learning [19].

AL constitute as a stage for the peer-to-peer education model, defined in the pedagogic process in which students act as a facilitator of the learning process to other students. Although there are controversies [20], this methodology could be used as another teaching strategy [21, 22]. The positive aspects include the preparation of future physicians for their roles in health education, the establishment and development of relationships and guidance. Besides that AL are less threatening and more motivating environment than traditional models of learning [23, 24]. In addition, it assists with preparation for future learning (PFL) and is very practical. It enables the student to learn new informations and correlate these with previous experiences, demonstrating innovation and flexibility to problem- solving. It also enables the student to unlearn obsolete information [19].

In Oncology AL, a pedagogical program classifies cancer knowledge into several aspects: epidemiology, molecular aspects, diagnostic and staging methods, treatment and the follow up of the most prevalent neoplasm, with strategies of prevention and screening.

### Management and leadership

The crucial role of leadership in medical education is well documented [25, 26]. Although academic leadership faces challenges (as organizational issues, mismatch between authority and responsibilities, a boss-centred culture and low motivation [26], students find in Academic Leagues an important scenario of learning and practice. The management of AL is elected amongst student members each year. Its role is to organize the process, set timelines, develop projects and mobilize students’ participation. This mobilization is guaranteed because students feel part of the project as they have an active voice and can provide feedback and set course [9]. Thus, it represents excellent opportunities for the development of individual and shared responsibility, promoting teamwork and enhancing skills such as decision-making and leadership. In fact, it is essential that the physician not only has effective knowledge of healthcare equipment, procedures and practices but also the capacity make decisions and administer appropriate use of healthcare. Finally, doctors must have excellent communication skills and be able to manage and administer both the workforce and material resources and information, acting as entrepreneurs, managers and leaders.

### Entrepreneurship

As AL activities are managed by students, they are able to hone entrepreneurial skills, acquire knowledge in the marketing management field and in processes in the field of administration. Generally, AL emerge as an answer to a problem (usually, a gap in the curriculum of medical schools). Students devise work strategies, dealing with all problems inherent with creation, implementation and monitoring such as planning, finding a venue for meetings, lack of financial resources and the development of a final product (which include courses or, most often, health education activities). Participating in a league, students learn how to handle and resolve unforeseen problems on a daily basis. This experience is important for planning both the personal and professional life dealing with aspects such as office management, changing schedules and management of income, resources and ideas [27].

### Innovation

Usually, AL use digital media as a tool of teaching and learning which includes discussion forums, texts, videos and educational material sharing. In fact, recent publications demonstrate the increasing use of these resources for educational purposes [28]. In the present study, analysis show that the majority of Oncology leagues have websites or profiles in social networks, many without public restriction. Once available on internet, these materials often reach laymen or are developed by students who wish to embark upon a course in health education. For example, in our research we found that many web pages showed information about cancer prevention like healthy lifestyles and how to combat smoking. Whilst many divulged information about clinical signs and the importance of early diagnosis and some recruited people for bone marrow donation. We also found records about health education in non-scientific literature, such as radio programs and interviews, electronic newsletters and newspapers.

### Health education

From an entrepreneurial perspective, health education is the main product developed by AL through popular education activities that can occur in several ways. Thus, we can observe a potential role that is surpasses professional qualification and allows student interaction with the population. Through these extra-curricular activities cancer prevention is highlighted in many ways, such as the distribution of education materials prepared by students themselves or outdoor activities on beaches, in parks and in elementary schools. It was demonstrated that interventional strategies such as education workshops, mass marketing, education materials and information advertised on social network could increase the number of people who regularly undertake screening for cancer [29]. Escoffery et al. [12] present in a review literature study how some activities could be used for cancer prevention and screening. Of the five main activities groups mentioned, all are continuously performed in AL, being planned and executed by undergraduate medical students: (1) health fairs involving the general public, for example, smoking cessation sessions, with distribution of educational material and application of specific tests questionnaires; (2) charity events with non-governmental organizations; (3) cultural events such as awareness campaigns about donation and registration with bone marrow banks; (4) special days mobilizing the population for a cancer cause, for example, sponsored walking events for the prevention of breast cancer; (5) theatre plays about cancer prevention for children and adolescents.

### Construction of citizenship

As a result of multiplicity of skills and competencies that students acquire and refine participating in AL, values exceed mere professional qualification. Personal development is stimulated, allowing the doctor have a multi-dimensional role not only limited to technique and science. They are able to engage and contextualize with the reality of the general public, acting as agents in the mobilization and social transformation, developing citizenship.

## Conclusion

In Brazil, the number of AL in medical schools is important and the impact of its activities should be discussed and continually evaluated. It is an initiative that deserves to be shared as another model for teaching and learning in Medical Education, to be authentic, designed, created and managed by students. In addition, AL are able to develop skills and competencies that are rarely stimulated whilst studying the traditional curriculum. This has a positive effect on professional training, community approach through prevention strategies, and development of the student on a personal level permitting a dynamic, versatile and attentive outlook to their social role. For Oncology AL, this encourages more skilled care to patients and more effective policies for cancer control.
